# Retraction: The impact of horse purslane (*Trianthema portulacastrum* L.) infestation on soybean [*Glycine max* (L.) Merrill] productivity in northern irrigated plains of Pakistan

**DOI:** 10.1371/journal.pone.0277580

**Published:** 2022-11-16

**Authors:** The PLOS ONE Editors

The *PLOS ONE* Editors retract this article [1] because it was identified as one of a series of submissions for which we have concerns about authorship, competing interests, and peer review. We regret that the issues were not addressed prior to the article’s publication.

KM, MS, NS, HuR, TAY, AW, MH, MBA, MWY, and SF did not agree with the retraction. MA and TAA either did not respond directly or could not be reached.
